# Supplementary material for: The Effect of Manufacture Process on Mechanical Properties and Burning Behavior of Epoxy-Based Hybrid Composites
Source: Materials (Basel). 2022 Jan 1;15(1):301. doi: 10.3390/ma15010301 (PMC8746176; doi:10.3390/ma15010301)
Supplement: Supplementary file 1 [file materials-15-00301-s001.zip › materials-1477754-supplementary.pdf]

materials

MDPI

Supplementary Material

The Effect of Manufacture Process on Mechanical Properties and Burning Behavior of Epoxy-Based Hybrid Composites

Kamila Sałasińska [1,2,*], Peteris Cabulis [3], Mikelis Kirpluks [4], Andrejs Kovalovs [3], Paweł Kozikowski [2], Mateusz Barczewski [5], Maciej Celiński [2], Kamila Mizera [2], Marta Gałecka [1], Eduard Skukis [3], Kaspars Kalnins [3], Ugis Cabulis [4] and Anna Boczkowska [1]

[1] Faculty of Materials Science and Engineering, Warsaw University of Technology, Wołoska 141, 02-507 Warsaw, Poland; sgalecki44@gmail.com (M.G.); anna.boczkowska@pw.edu.pl (A.B.)
[2] Department of Chemical, Biological and Aerosol Hazards, Central Institute for Labour Protection—National Research Institute, Czerniakowsa 16, 00-701 Warsaw, Poland; pawel.kozikowski@ciop.pl (P.K.); maciej.celinski@ciop.pl (M.C.); kamila.mizera@ciop.pl (K.M.)
[3] Institute of Materials and Structures, Riga Technical University, 6b Kipsalas St., LV-1048 Riga, Latvia; peteris@ritols.lv (P.C.); andrejs.kovalovs@rtu.lv (A.K.); edskukis@gmail.com (E.S.); kaspars.kalnins@rtu.lv (K.K.)
[4] Polymer Laboratory, Latvian State Institute of Wood Chemistry, 27 Dzerbenes St., LV-1006 Riga, Latvia; mkirpluks@kki.lv (M.K.); cabulis@kki.lv (U.C.)
[5] Institute of Materials Technology, Poznan University of Technology, Piotrowo 3, 61-138 Poznan, Poland; mateusz.barczewski@put.poznan.pl
* Correspondence: kamila.salasinska@pw.edu.pl

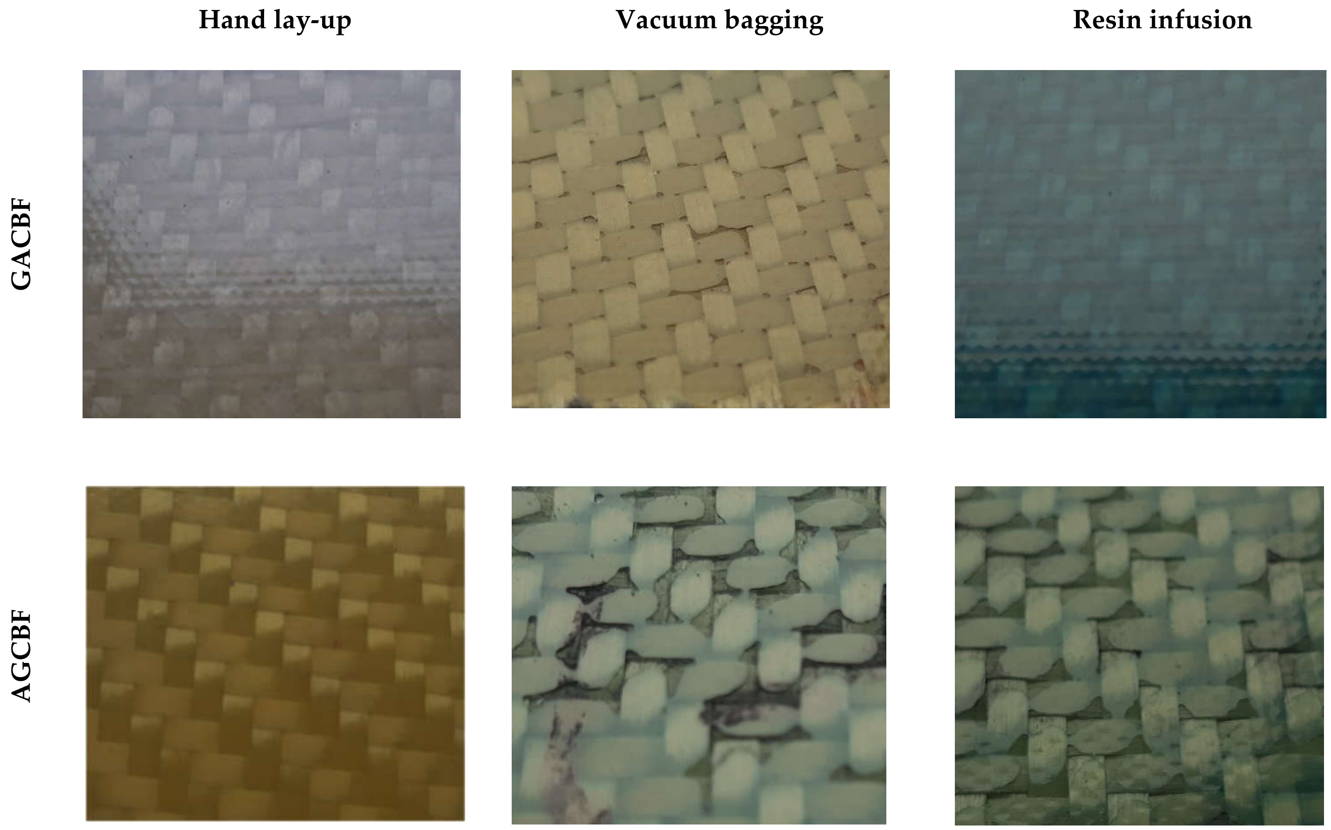

Figure S1. Photographs of samples surface made by hand lay-up, vacuum bagging and resin infusion methods.

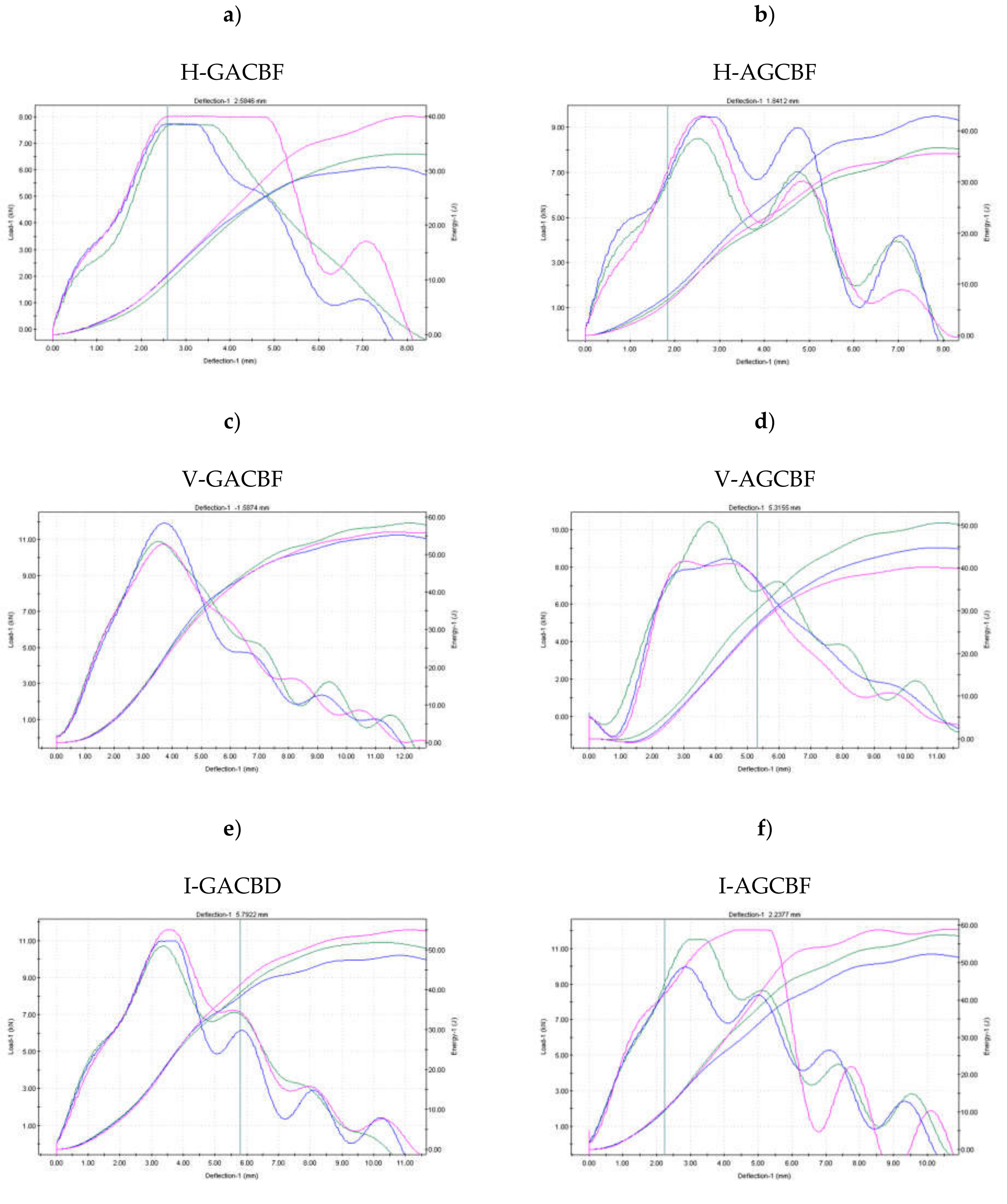

Figure S2. Force and energy vs. deflection plots measured by low-speed impact test for hybrid composites.
